# Citizen science for research in public health: perspective

**DOI:** 10.3389/fpubh.2025.1594293

**Published:** 2025-07-30

**Authors:** Hatem H. Alsaqqa, Abdallah Alwawi

**Affiliations:** ^1^Deanship of Scientific Research, Al-Quds University, Jerusalem, Palestine; ^2^Palestinian Ministry of Health, Gaza, Palestine; ^3^Health Professions Faculty-Department of Nursing, Al Quds University, Jerusalem, Palestine

**Keywords:** citizen science, policy, public engagement, public health, research capacity

## Abstract

In order to enhance health and lessen inequities, public engagement strategies that unite various stakeholders to jointly identify issues and develop alternatives are acknowledged as an essential tactic to support research-informed policy and implementation. Through the use of citizen science methods, researchers can collect and analyze more information and make research more cost-effective by enlisting members of the public as participants. Research on health has already been greatly impacted by the vast and unique data sets acquired through citizen science methodologies. Engaging community viewpoints into public health research and policy-making has proven difficult. Thus, research capacity must be developed to allow academics and policy-making and implementation stakeholders to meaningfully interact with the public. A fundamental tenet of public health is involving the community, which is seen as being vital in ensuring that initiatives and regulations designed for enhancing health and welfare that are pertinent to local needs and make the most from scarce resources.

## Introduction

In an era marked by growing demands for inclusive and community-driven solutions to health challenges, the role of citizen science in public health is beginning to attract meaningful attention. According to Haklay et al. (1), citizen science approaches are a way to dynamically involve the public in scientific research, such as in data collection and analysis and research design. Despite having its roots in the natural sciences, citizen science techniques are being utilized increasingly to engage the public in the collection and interpretation of data in order to solve a variety of public health concerns (2).

According to Franzoni et al. (3), citizen science is broadly defined as “public engagement and cooperation in scientific research.” It involves members of the public (also known as “citizen scientists”) in a variety of research activities, such as formulating research questions, creating project methodologies, gathering and analyzing data, and arguing, understanding and sharing research findings. One characteristic that sets citizen science apart is that it involves participants as active participants in the research procedure (3). Citizen participation ranges from independently led community initiatives to active collaboration in researcher-driven projects (4). However, ethics, the intricacy of the health domain, and the overlap in roles where the researcher is occasionally the subject of research were the primary concerns identified as being unique to citizen science in health (5).

In order to formulate research questions, gather and evaluate data, interpret findings, generate new discoveries, and create technologies and applications, citizen science leverages the combined strength of communities (6). Citizen science has the capacity to produce vast amounts of data and involves citizens in addressing and providing answers to complicated environmental and societal challenges. Additionally, according to Sherbinin et al. (7), citizen science is a crucial instrument for democratizing science and promoting fair and available access to scientific data and knowledge.

Moreover, citizen science application in public health is seen as a viable approach to building more equitable and healthy communities and neighborhoods (8). According to the philosophy of citizen science (9), citizens are viewed as subjects who aim to gain from their study since it advances their own personal growth and self-determination.

Although academic researchers have traditionally spearheaded citizen science in public health and health promotion to date (2, 10), policy and practice stakeholders are becoming more interested in these methods as an adjunct to including the public in their work (11). Stakeholders in policy and practice organizations that oversee health and wellbeing, such as local health districts, councils, and health promotion agencies, are becoming more involved in implementing these strategies in their work (12).

However, enhancing the use of evidence is a continuous goal of capacity building in the public health sector. Evidence that is co-produced with knowledge users (such as public health practitioners and policymakers and patients) has a higher chance of being incorporated into practice and policy, according to years of work to support “research translation.” The way innovation occurs in the health care ecosystem is anticipated to be revolutionized by citizen involvement, given the increasing availability of digital solutions that provide real-time and home-based data collecting (13).

Citizen Science in public health aims to accomplish the following main goals: create new knowledge about the viability, effects, and possible drawbacks of citizen science approaches in practice and policy contexts; raise awareness and acceptance of citizen science; facilitate the exchange of knowledge and perspectives, and create a network of interested parties (14).

Nevertheless, using a continuum of citizen scientists’ degree of participation in the study process is one method of categorizing citizen science methodologies. These include “contributory” approaches, where citizen scientists primarily assist with data collection and/or analysis; collaborative approaches, where citizens can participate in different phases of the research process alongside researchers; and co-created citizen-led approaches which are distinguished by increased citizen scientist leadership and control (2). The added value of community engagement in public health research should be better understood, so more capacity has to be provided for academics to disclose this impact. In turn, health researchers need to listen and open up to citizens’ demands and become more democratic, transparent, and aligned with society’s demands.

## Health research capacity building and context

One characteristic that sets citizen science apart is that it involves participants as vigorous participants in the scientific process. Increased research capacity, the inclusion of community viewpoints on issues and solutions, and enhanced public awareness and reception of health-improving initiatives are some possible advantages of citizen science approaches. According to a recent scoping review, citizen science methods have been applied to: identify issues from the viewpoint of the community; prioritize and generate alternatives; create, test, and/or assess interventions; and/or increase community capacity (2).

Capacity building can be done with individuals, groups, organizations, and/or communities. It is “an approach for fostering the creation of sustainable skills, organizational structures, resources, and a dedication to advancing health and other sectors.” Thus, it is anticipated that more research in this field will reveal ways that well-established citizen science techniques could enhance the caliber and applicability of public participation in health research.

However, the 21st century has seen a sharp rise in citizen science participation due to participatory democracy, societal and technical advancements, and other factors. Crucially, technology has made it possible for scientific data to be gathered and processed on a massive scale and for scientific information and discoveries to be widely shared (15). Accomplishing universal high-quality and safe health care coverage requires developing and enhancing health research capacity (16). By enabling driven healthcare professionals and researchers to produce evidence and implement results in a way that is pertinent to their local context, the enhanced research capacity may foster the mixture of research and clinical practice (17).

The multi-level process, research capacity building entails funding and assisting individuals, groups, organizations, and networks of organizations in order to raise demand for research, support researchers’ ability to carry out studies, and facilitate the efficient application of findings. Investing in organizational, technological, and human resources at different organizational levels is part of the complicated process of building health research capacity (18).

Furthermore, research capacity entails helping researchers locate and evaluate existing literature, come up with research ideas, gather and evaluate qualitative and quantitative data, write and present findings, and locate money, mentorship, and time to carry out research (19). Increased funding, the development of more skilled researchers, support for regional and global long-term collaborations, and other administrative advancements in executive and supervisory mechanisms are all necessary to reinforce research capacity at the facility and systems levels (20).

Nonetheless, one strategy to guarantee that these contextual elements are adequately included in the research design and to promote the sustainability of interventions is to involve the public at every stage of the research process (21). When working with rural or remote communities that are farther away from researchers and where contextual considerations are less widely recognized, it may be especially effective to involve cultural or knowledge advisors as a way to help researchers recognize community situation and find individuals doing related work on the ground (22). However, a number of constraints including a lack of funding, institutional support and infrastructure, research expertise, and know-how, make it difficult for researchers to plan and carry out studies that are essential to their requirements. Therefore, in order to guide national initiatives aimed at developing reinforced health systems, it is vital to understand the contextual elements that either support or hinder efforts to create research capacity.

## Involving members and social practice

Interest in quantifying the impact of public participation in health research has increased as it has become a global standard for the design, execution, and diffusion of health research. Supporters of public participation in health research are eager to see its advantages proven while detractors hope their suspicions will be validated, So far, others want to learn more about whether and how community involvement disturbs research actions and outcomes, contributors, and any varied societal impacts (23).

Yet, studies of stakeholder perspectives and systematic reviews (24) have addressed the global literature on how to evaluate the impact of public involvement. In order to assess the nature and impact of public involvement in health research, at least 65 frameworks have been created as summarized in a current systematic review (25). Other studies have also advanced practical guidelines for researchers that include the estimation of the impact of public involvement in their research.

When taken as a whole, these studies provide a strong body of data supporting several claims about the benefits of public participation in health research. For instance, they have noted how public participation can improve clinical trial recruitment (26), make research more user-relevant and suitable (26). It also assist in developing research questions and reshaping study design (27), and offer insights to direct and foster analysis.

However, there are regular and broad calls for more reliable techniques and tools to collect and assess effect (28). A sizable amount of the available data regarding impact is regarded as anecdotal and poor (29). Some groups are starting to recognize the need of ongoing reflection as part of the research process and are moving away from an instrumental view of public involvement as an “intervention” with a quantifiable effect (30).

Staley and Barron (29) view public involvement as a social practice of communication and education between the public and researchers: an objective in and of itself, rather than just a means to an end (at worst, superficially assessed as “bums on seats”). When, why, and with whom the dialog occurs or does not occur are some of the questions that critical public engagement research should address to examine the richness and complexity of this interaction (31).

Nevertheless, the patient and public involvement tools may assist patients and members of the public in thinking about their participation as research partners in the following ways: (i) to make it easier for patients to be included as research partners and to indorse to the study; (ii) to recognize projects from the perspective of patients and partners from that of researchers; (iii) to ensure that partners and researchers esteem each other’s contributions and close information; (iv) to support the patient partners’ capacity to collaborate and communicate with the researcher and their assembly; and (v) to educate patient partners on research methods and procedures to foster some comprehension of the course among the patient associates (32).

## Legitimacy of community perspectives

As the public is actively intricated in all phases of the research-to-policy process(including urgency setting, research design, policy design, implementation, and evaluation) that can create successful public health interventions that tackle unresolved public priorities and are acceptable to the public. This calls for enhancing the capacity of researchers and public health professionals to interact with the general public and different populations in an appropriate manner.

Therefore, it is crucial to encourage open communication with participants (including discussion of the broader social determinants of health), co-design of technologies, careful evaluation of the permitting context, and evocative engagement with susceptible entities and underserved groups. It is necessary especially when creating wide-ranging research and plans to address public health significances and health disparities. While guaranteeing legitimacy and increased confidence in public health technologies, these tactics may promote public agency and data sharing for research objectives (33).

## Discussion

Individuals have the right to energetically take part in their healthcare and should have appropriate, high-quality, culturally related information, backing, and services that empower people to learn about and take part in their health in various ways. Health funders and deliverers are gradually attempting to quantity and apply principles like collective decision-making and person-centered care, which are recognized as essential components of a functioning health system (34); patient experience-led enhancement (35); health literacy (36); and the codesign of health services, strategy, and research.

The authors describe these terms together in this study as experiences with, or endeavors to enhance, “health communication and participation.” People frequently have less than ideal experiences with health communication and participation, even with significant efforts. Poor communication and insufficient patient involvement in their health have an impact on healthcare value and safety, in addition to the evident ethical requirements. Developing research priorities with interested parties is believed to decrease research waste and match research with the demands of those it impacts.

However, with the general public, putting into practice hands-on, acceptable, and sustainable measures is frequently challenging, and establishing environments that promote health necessitates the support and upsurge of a diversity of sponsors. Involving the public in the planning, execution, and assessment of prevention programs is a known way to guarantee that health policies and programs appropriately represent the needs, queries, and viewpoints of communities (37). The necessity of inclusive and cooperative methods for scientific research (38) and decision-making that impacts public health and well-being (39) is becoming increasingly apparent.

Despite having their roots in the natural sciences, citizen science methodologies have expanded quickly in recent years to include public health (40). In addition to sharing similarities with other methods of involving the public in research (such as community-based participatory research and participatory action research), citizen science expands upon longstanding backgrounds of public participation in health promotion.

With the stated objective of minimizing personal harm and maximizing public value, research and biomedical ethics are an essential component of medicine and health research (41). Additional ethical frameworks with an emphasis on societal justice are required in public health research, where groups and communities, rather than individuals, are at the center (42). The relationships among participants in citizen science projects should be closely examined by ethicists interested in care ethics, who should ask questions about how good are these relationships and if the participants need to be recognized and if the care being provided is necessary and is good care (43).

Nonetheless, the evidence basis for community-centered population health research is unbalanced, and community members (or the public) are less frequently included on research staffs, while being a significant information user-group (44). Research teams will be better able to operationalize community member participation in health research in a means that provisions and takes into consideration the needs, preferences, and characteristics of specific groups of people.

That can generate best preparation procedures if they have a regional awareness of the concepts and tactics for community participation.

Participants frequently voiced negative opinions about researchers or decision-makers in the health system traveling from larger cities, gathering information or holding tokenistic consultations on changes to the health system, and then departing without offering sufficient input. A lack of perceived ownership may be the cause of these frequently reported sentiments of dissatisfaction with the past involvement in research.

Contradictory to this negative view, the PriCARE research program; a multiple-case embedded mixed-methods study design that has been out since 2018 in five Canadian provinces, is one instance of patient involvement in health research (45). The aim is to investigate how a case management (CM) intervention is applied in primary care clinics across Canada for people who often utilize health care services and have complex care needs and chronic illnesses. The goals are to determine what supports and hinders the use of CM in primary care clinics across Canada (46).

One excellent example of finding a compromise between developing a more patient-oriented tool and maintaining the instruments, which are prevalidated standardized questionnaires, is the creation of patient-oriented recommendations for administering the patient questionnaires. Academic researchers notably brought attention to time restrictions associated with the study program, as they occasionally felt that they lacked sufficient time to devote to patient participation. Patient partners stated that they might have saved time and expedited the creation of the patient questionnaire by participating in the first stages of data collecting, such as choosing and identifying the instruments to be used in the questionnaire (47).

However, by pointing out problems that the research team would have otherwise overlooked, patient partners believed that by expressing their viewpoints, the validity of the study’s findings may eventually be improved. Patient partners provided input that helped create better user-friendly research instruments. In their reflections on these contributions, academic researchers pointed out that the lived experiences of patient partners offered valuable insights into improving patients’ rapport. Both groups recognized the value and positive impact of patient engagement in the program in terms of improving the relevance of research and the applicability of results (47).

Nevertheless, a research methodology known as “integrated knowledge translation” promotes the co-production of research with information user team associates, who are generally characterized as individuals or groups that stand to gain from, or may apply, research conclusions in practice or to inform decision-making (44). Research funders, healthcare professionals, decision-makers in the health system, groups of supporters, patient organizations, and/or members of the general public are examples of knowledge users. Yet, in research, community empowerment and ownership are best accomplished through genuine collaborations, which necessitate a power shift from researchers to communities and the development of researcher trust (48).

Considering and reporting on these strategies for ensuring inclusion as well as diversity, is important for citizen science developments, and it is crucial to engage diverse perspectives in order to address discriminations in population health, as citizen science provides occasions to intensify participation of population assemblies that are usually excepted from research and decision-making processes.

## Data Availability

The original contributions presented in the study are included in the article/supplementary material, further inquiries can be directed to the corresponding author.

## References

[1] Haklay M Dörler D Heigl F Manzoni M Hecker S Vohland K. What is citizen science? The challenges of definition. Sci Citizen Sci. (2021) 13:34–51. doi: 10.1007/978-3-030-58278-4_2#DOI

[2] Marks L Laird Y Trevena H Smith BJ Rowbotham S. A scoping review of citizen science approaches in chronic disease prevention. Front Public Health. (2022) 10:743348. doi: 10.3389/fpubh.2022.743348, PMID: 35615030 PMC9125037

[3] Franzoni C Poetz M Sauermann H. Crowds, citizens, and science: a multi-dimensional framework and agenda for future research. Ind Innov. (2022) 29:251–84. doi: 10.1080/13662716.2021.1976627

[4] King AC Winter SJ Sheats JL Rosas LG Buman MP Salvo D. Leveraging citizen science and information technology for population physical activity promotion. Transl J Am Coll Sports Med. (2016) 1:30–44. doi: 10.1249/TJX.0000000000000003, PMID: 27525309 PMC4978140

[5] Remmers G Wildevuur S Assens SM Tzovaras BG De Groot M Torrents EG. Citizen science for health: an international survey on its characteristics and enabling factors. Citizen Sci Theory Pract. (2024) 9:1–6. doi: 10.5334/cstp.693

[6] United States Environmental Protection Agency. (2020). Citizen science for environmental protection. Available online at: https://www.epa.gov/citizen-science (Accessed on 10 November 2020)

[7] Sherbinin A Bowser A Chuang TR Cooper C Danielsen F Edmunds R. The critical importance of citizen science data. Front Clim. (2021) 3:650760. doi: 10.3389/fclim.2021.650760

[8] Den Broeder L Devilee J Van Oers H Schuit AJ Wagemakers A. Citizen science for public health. Health Promot Int. (2018) 33:505–14. doi: 10.1093/heapro/daw086, PMID: 28011657 PMC6005099

[9] Smith K. Descartes’ Theory of Ideas, Metaphysics Research Lab, Stanford University, Stanford, CA. (2021).

[10] Borda A Gray K Downie L. Citizen science models in health research: an Australian commentary. Online J Public Health Info. (2019) 11:e23. doi: 10.5210/ojphi.v11i3.10358 PMC6975539 31993110

[11] Rowbotham S Laird Y Marks L Walker P Pontifex K Sobhan A. Building capacity to apply citizen science approaches in policy and practice for public health: protocol for a developmental evaluation of four stakeholder-led projects. University of Tasmania. Journal contribution. (2022). Available at: https://hdl.handle.net/102.100.100/550066

[12] Rowbotham S Marks L Tawia S Woolley E Rooney J Kiggins E. Using citizen science to engage the public in monitoring workplace breastfeeding support in Australia. Health Promot J Austr. (2022) 33:151–61. doi: 10.1002/hpja.476, PMID: 33690925

[13] Ciasullo MV Carli M Lim WM Palumbo R. An open innovation approach to co-produce scientific knowledge: an examination of citizen science in the healthcare ecosystem. Eur J Innov Manag. (2022) 25:365–92. doi: 10.1108/EJIM-02-2021-0109

[14] Rowbotham S Walker P Marks L Irving M Smith BJ Laird Y. Building capacity for citizen science in health promotion: a collaborative knowledge mobilisation approach. Res Involvement Engag. (2023) 9:36. doi: 10.1186/s40900-023-00451-4, PMID: 37254184 PMC10228445

[15] Wiggins A Crowston K. Surveying the citizen science landscape. First Monday. (2015) 20:1–5. doi: 10.5210/fm.v20i1.5520, PMID: 40395440

[16] Hanney SR González-Block MA. Organising health research systems as a key to improving health: the world health report 2013 and how to make further progress. Health Res Policy Syst. (2013) 11:1–5. doi: 10.1186/1478-4505-11-47, PMID: 24341347 PMC3878484

[17] Mansour R Naal H Kishawi T Achi NE Hneiny L Saleh S. Health research capacity building of health workers in fragile and conflict-affected settings: a scoping review of challenges, strengths, and recommendations. Health Res Policy Syst. (2021) 19:84. doi: 10.1186/s12961-021-00725-x, PMID: 34022883 PMC8140497

[18] Cooke J Gardois P Booth A. Uncovering the mechanisms of research capacity development in health and social care: a realist synthesis. Health Res Policy Syst. (2018) 16:1–22. doi: 10.1186/s12961-018-0363-4, PMID: 30241484 PMC6150992

[19] Busse CE Anderson EW Endale T Smith YR Kaniecki M Shannon C. Strengthening research capacity: a systematic review of manuscript writing and publishing interventions for researchers in low-income and middle-income countries. BMJ Glob Health. (2022) 7:e008059. doi: 10.1136/bmjgh-2021-008059, PMID: 35165096 PMC8845213

[20] Bowsher G Papamichail A El Achi N Ekzayez A Roberts B Sullivan R. A narrative review of health research capacity strengthening in low and middle-income countries: lessons for conflict-affected areas. Glob Health. (2019) 15:1–3. doi: 10.1186/s12992-019-0465-y, PMID: 30914049 PMC6434620

[21] Jagosh J Macaulay AC Pluye P Salsberg JO Bush PL Henderson JI. Uncovering the benefits of participatory research: implications of a realist review for health research and practice. Milbank Q. (2012) 90:311–46. doi: 10.1111/j.1468-0009.2012.00665.x, PMID: 22709390 PMC3460206

[22] Pinsoneault LT Connors ER Jacobs EA Broeckling J. Go slow to go fast: successful engagement strategies for patient-centered, multi-site research, involving academic and community-based organizations. J Gen Intern Med. (2019) 34:125–31. doi: 10.1007/s11606-018-4701-6, PMID: 30353249 PMC6318189

[23] Friesen P Lignou S Sheehan M Singh I. Measuring the impact of participatory research in psychiatry: how the search for epistemic justifications obscures ethical considerations. Health Expect. (2021) 24:54–61. doi: 10.1111/hex.12988, PMID: 31854081 PMC8137501

[24] Brett JO Staniszewska S Mockford C Seers K Herron-Marx S Bayliss H The PIRICOM study: a systematic review of the conceptualisation, measurement, impact and outcomes of patients and public involvement in health and social care research. Coventry, UK: University of Warwick (2010)

[25] Greenhalgh T Hinton L Finlay T Macfarlane A Fahy N Clyde B. Frameworks for supporting patient and public involvement in research: systematic review and co-design pilot. Health Expect. (2019) 22:785–801. doi: 10.1111/hex.12888, PMID: 31012259 PMC6737756

[26] Staley K Kabir T Szmukler G. Service users as collaborators in mental health research: less stick, more carrot. Psychol Med. (2013) 43:1121–5. doi: 10.1017/S0033291712001663, PMID: 22850532 PMC3642719

[27] Wykes T. Great expectations for participatory research: what have we achieved in the last ten years? World Psychiatry. (2014) 13:24–7. doi: 10.1002/wps.20086, PMID: 24497239 PMC3918010

[28] Madden M Speed E. Beware zombies and unicorns: toward critical patient and public involvement in health research in a neoliberal context. Front Sociol. (2017) 2:7. doi: 10.3389/fsoc.2017.00007

[29] Staley K Barron D. Learning as an outcome of involvement in research: what are the implications for practice, reporting and evaluation? Res Involvement Engag. (2019) 5:14. doi: 10.1186/s40900-019-0147-1, PMID: 30915234 PMC6416961

[30] Hickey G Brearley S Coldham T Denegri S Green G Staniszewska S. Guidance on co-producing a research project. Southampton: Involve (2018).

[31] Tsoukas H. Don't simplify, complexify: from disjunctive to conjunctive theorizing in organization and management studies. J Manag Stud. (2017) 54:132–53. doi: 10.1111/joms.12219

[32] Hewlett S Wit MD Richards P Quest E Hughes R Heiberg T. Patients and professionals as research partners: challenges, practicalities, and benefits. Arthritis Care Res: Official J American College Rheumatol. (2006) 55:676–80. doi: 10.1002/art.22091 16874772

[33] The Health Foundation & FrameWorks UK. How to talk about the building blocks of health: A communications toolkit for people working in public health. (2022) (Accessed March, 2025).

[34] World Health Organization. Framework on integrated, people-centred health services. Geneva: World Health Organization (2016).

[35] Raleigh V Thompson J Jabbal J Graham C Sizmur S Coulter A. Patients’ experience of using hospital services. London, UK: King’s Fund (2015).

[36] Brega A. AHRQ health literacy universal precautions toolkit. 2nd ed. Rockville, MD: Agency for Healthcare Research and Quality (2015).

[37] World Health Organization. Community engagement for quality, integrated, people-centred and resilient health services. (2020). (Accessed March, 2025).

[38] Lloret J Abós-Herràndiz R Alemany S Allué R Bartra J Basagaña M. The roses ocean and human health chair: a new way to engage the public in oceans and human health challenges. Int J Environ Res Public Health. (2020) 17:5078. doi: 10.3390/ijerph17145078, PMID: 32674437 PMC7400534

[39] Morrison N Barns S Dunshea A Paine G Pry J Sajan J. Making Healthy Places: NSW Built Environment Practitioners’ Perspectives on Place-Making Opportunities That Help Deliver Health and Wellbeing Outcomes. Liverpool, NSW: Marudulu Budyari Gumal (2021). doi: 10.52708/LCWA1416

[40] Kullenberg C Kasperowski D. What is citizen science?–a scientometric meta-analysis. PLoS One. (2016) 11:e0147152. doi: 10.1371/journal.pone.0147152, PMID: 26766577 PMC4713078

[41] McNair L. Ethical and regulatory oversight of clinical research: the role of the institutional review board. Exp Biol Med. (2022) 247:561–6. doi: 10.1177/15353702221078216, PMID: 35172623 PMC9014527

[42] Ballantyne A. Adjusting the focus: a public health ethics approach to data research. Bioethics. (2019) 33:357–66. doi: 10.1111/bioe.12551, PMID: 30667080

[43] Remmers G Hermann J Siebrand E Van Leersum CM. Mind the relationship: a multi-layered ethical framework for citizen science in health. Etica e Politica. (2023) 25:171–96. doi: 10.13137/1825-5167/35356

[44] Canadian Institute of Health Research. Knowledge user engagement. (2016). Available online at: http://www.cihr-irsc.gc.ca/e/49505.html. (Accessed 29 November 2019).

[45] Hudon C Chouinard MC Aubrey-Bassler K Burge F Doucet S Ramsden VR. Case management in primary care for frequent users of healthcare services with chronic diseases and complex care needs: an implementation and realist evaluation protocol. BMJ Open. (2018) 8:e026433. doi: 10.1136/bmjopen-2018-026433, PMID: 30478129 PMC6254422

[46] Danish A Chouinard MC Aubrey-Bassler K Burge F Doucet S Ramsden VR. Protocol for a mixed-method analysis of implementation of case management in primary care for frequent users of healthcare services with chronic diseases and complex care needs. BMJ Open. (2020) 10:e038241. doi: 10.1136/bmjopen-2020-038241, PMID: 32487584 PMC7265033

[47] Béland S Lambert M Delahunty-Pike A Howse D Schwarz C Chouinard MC. Patient and researcher experiences of patient engagement in primary care health care research: a participatory qualitative study. Health Expect. (2022) 25:2365–76. doi: 10.1111/hex.13542, PMID: 35593113 PMC9615076

[48] Salsberg J Macridis S Garcia Bengoechea E Macaulay AC Moore S KSDPP School Travel Planning Committee. Engagement strategies that foster community self-determination in participatory research: insider ownership through outsider championship. Fam Pract. (2017) 34:336–40. doi: 10.1093/fampra/cmx001, PMID: 28334802

